# Aesthetic Perception of Line Patterns: Effect of Edge-Orientation Entropy and Curvilinear Shape

**DOI:** 10.1177/2041669520950749

**Published:** 2020-09-30

**Authors:** Sarah Stanischewski, Carolin S. Altmann, Anselm Brachmann, Christoph Redies

**Affiliations:** Experimental Aesthetics Group, Institute of Anatomy, Jena University Hospital, University of Jena School of Medicine

**Keywords:** experimental aesthetics, visual preference, curvilinearity, luminance edges, curved/angular shape, texture, extraclassical receptive field, visual cortex

## Abstract

Curvilinearity is a perceptual feature that robustly predicts preference ratings for a variety of visual stimuli. The predictive effect of curved/angular shape overlaps, to a large degree, with regularities in second-order edge-orientation entropy, which captures how independent edge orientations are distributed across an image. For some complex line patterns, edge-orientation entropy is actually a better predictor for what human observers like than curved/angular shape. The present work was designed to disentangle the role of the two features in artificial patterns that consisted of either curved or angular line elements. We systematically varied these patterns across two more dimensions, edge-orientation entropy and the number of lines. Eighty-three participants rated the stimuli along three aesthetic dimensions (*pleasing*, *harmonious*, and *complex*). Results showed that curved/angular shape was a stronger predictor for ratings of *pleasing* and *harmonious* if the stimuli consisted of a few lines that were clearly discernible. By contrast, edge-orientation entropy was a stronger predictor for the ratings if the stimuli showed many lines, which merged into a texture. No such differences were obtained for complexity ratings. Our findings are in line with results from neurophysiological studies that the processing of shape and texture, respectively, is mediated by different cortical mechanisms.

One of the central questions in experimental aesthetics is how objective image properties affect the human preference for particular visual stimuli. For example, the founder of experimental aesthetics, Gustav Theodor Fechner (1801–1887), studied whether particular size proportions and other characteristics make simple objects more pleasing to human observers (Fechner, 1876). Understanding and predicting human aesthetic preferences is a hot topic not only in vision research but also in marketing studies and product design (Landwehr et al., 2012; Wang et al., 2019).

It is generally agreed that aesthetic experience results from an interplay of three fundamentals: perception, cognition, and emotion (Chatterjee & Vartanian, 2014; Graf & Landwehr, 2015; Jacobsen, 2006; Redies, 2015). It has been argued that some of the mechanisms that mediate aesthetic experience are universal among human observers (Bell, 1914) because they relate to common perceptual, cognitive, and/or affective processes (Berlyne, 1974; Eysenck, 1940; Redies, 2015). In support of this notion, cross-cultural studies have provided evidence that humans share preferences for perceptual features such as complexity, symmetry, proportion, and contrast (for a comprehensive review, see Che et al., 2018). More recently, complex global image properties have been associated with visual preference (for a review, see Brachmann & Redies, 2017). Examples are a scale-invariant Fourier spectrum (Graham & Field, 2007; Redies et al., 2007), fractality (Taylor et al., 2011), or regularities in the distribution of luminance and color edges (Brachmann et al., 2017; Braun et al., 2013; Redies et al., 2017). Presumably, such physical stimulus features are processed in a bottom-up fashion by neural mechanisms that are fast and automatic.

The contribution of cognition to aesthetic experience involves cultural and individual aspects, such as the historical context, aesthetic concepts, artistic intentions, and expertise as well as the observer’s individual preferences (Danto, 1981; Graf & Landwehr, 2015; Jacobsen, 2006; Leder et al., 2004). Cognitive processing, which relies more strongly on top-down mechanisms, is slower and highly individual. It has been proposed that processing in the perceptual and cognitive channels occurs in parallel, but the two channels interact prior to reaching a conscious aesthetic experience (Redies, 2015).

The role of emotions in aesthetic experience has been less well studied (for reviews, see Mastandrea, 2015; Silvia, 2014). On one hand, emotions can be elicited during an aesthetic experience, for example, when viewing an artwork. On the other hand, emotions can modulate aesthetic experience.

A stimulus property that has a striking and robust impact on our aesthetic experience is the shape of objects. Confirming earlier observations (Hevner, 1935; Hogarth, 1753; Poffenberger & Barrows, 1924; Stratton, 1902), Bar and Neta (2006) found that human observers rated curved contours and figures as more pleasing than angular ones. They proposed that angularity reflects a potential danger; it is thus perceived as less attractive because it induces fear, for example, by sharp-edged objects, such as knives or thorns. Indeed, angular stimuli elicit higher amygdala activation in neuroimaging studies (Bar & Neta, 2006). However, Palumbo et al. (2015) studied approaching and avoiding reactions to curved and angular polygons and concluded that the preference for curved forms is due to an intrinsic liking of positive attributes of curvilinearity rather than to a perceived threat associated with angular shapes. For simple polygons, Bertamini et al. (2019) found that responses of human observers to curved shapes were faster than to angular ones in different discrimination tasks, suggesting that the human visual system processes curvature more efficiently.

The preference of curvilinear shapes has been confirmed in several studies (Bertamini et al., 2016; Blazhenkova & Kumar, 2018; Gómez-Puerto et al., 2016; Silvia & Barona, 2009), including a cross-cultural investigation (Gómez-Puerto et al., 2018). This preference is even found in great apes, but less pronounced than in humans (Munar et al., 2015). Observers prefer curvilinearity also in architecture (Grebenkina et al., 2018; Thömmes & Hübner, 2018; Vartanian et al., 2013) and commercial products (Westerman et al., 2012). Wang et al. (2019) showed that the preference of curvature can be subject to a framing effect. Specifically, they reported that hedonic (but not utilitarian) product type moderates the effect of typeface curvature on consumer’s product preferences. Last, but not least, individual differences in the extent of curvature preferences have been described (Cotter et al., 2017).

Grebenkina et al. (2018) studied artificial line patterns that consisted of either curved (smooth) or straight (angular) lines (their Experiment 6). In addition to line shape, their patterns differed systematically in edge-orientation entropy, which is a measure of how uniform edge orientations are distributed across the full spectrum of orientations in an image (first-order entropy), or how independent edge orientations are distributed across an image (second-order entropy; Geisler et al., 2001; Redies et al., 2017). In both cases, Shannon entropy serves as a measure for histogram uniformity. The focus of the present study is second-order entropy of edge orientations (henceforth abbreviated as Entropy). To calculate this value, the orientation of each edge element is related to the orientation of all other edge elements in the same image by pairwise comparison. For all edge pairs, the orientation differences are plotted in a histogram. Entropy of the resulting histogram is high if all orientation differences are equally likely to occur for all edge pairs at any position, that is, if edge orientations do not show regularities across an image, such as collinearity, parallelism, or cocircularity (Geisler et al., 2001). Redies et al. (2017) found that traditional artworks of different cultural backgrounds are characterized by high second-order edge-orientation entropy. Edge-orientation entropy should not be confused with Shannon entropy of gray level values, which is a widely used measure of image statistics that refers to the probability of encountering particular gray level values in an image (Kersten, 1987).

Because straight lines represent exactly one orientation and curved lines comprise many orientations, Entropy and curved/angular shape relate to each other. Grebenkina et al. (2018) reported that the two features share a large portion of predicted variance for preference ratings. For ratings of how pleasing and harmonious particular stimuli are, edge orientation entropy turned out to be a more powerful predictor than curvilinearity; in other words, shape was not the decisive feature that determined the aesthetic rating, but it was partially overridden by edge-orientation entropy. This finding is incongruent with the generally observed, robust preference for curved over angular shapes. In view of the partially overlapping predictive power of edge-orientation entropy and shape, the present study was designed to disentangle the individual contribution of the two measures to visual preference.

To analyze the effect of edge-orientation entropy and shape on visual preference, we studied artificial abstract patterns that consisted of either curved or angular lines. In addition, the lines were arranged so that the stimuli exhibited either a low or a high degree of second-order edge-orientation entropy. As a third variable in the experiment, we systematically varied the number of lines per image (5, 10, 20, or 40 lines; 2 × 2 × 4 design). We hypothesized that a small number of lines would strengthen the effect of line shape on the ratings because each individual line in the stimulus stands out more clearly. Conversely, a large number of lines cause individual lines to merge perceptually into an overall texture. We expected that, in this case, edge-orientation entropy would have a larger effect on aesthetic ratings than line shape, as shown previously (Grebenkina et al., 2018). Moreover, we hypothesized that a line pattern is preferred if edge-orientation entropy is high. Similar results have been obtained previously for several types of natural and artificial images that are aesthetically preferred (Grebenkina et al., 2018), including traditional artworks of different cultural backgrounds (Redies et al., 2017).

To cover different aspects of aesthetic perception, our participants rated the line patters along the dimensions of *pleasing*, *harmonious*, and *complex*. These rating terms are the dependent variables in our experiment; they are italicized throughout the remainder of the text. Previous studies revealed a difference between the terms *pleasing* and *harmonious* in the evaluation of aesthetic stimuli. Whereas *pleasing* describes the subjective emotional arousal that is elicited by an image (Cupchik & Gebotys, 1990), *harmonious* reflects more closely the pictorial structure of an image (Redies et al., 2015). Lyssenko et al. (2016) showed that the usage frequency of such structure-related terms correlates with statistical image properties.

Furthermore, we used the rating dimension *complex*, which depends primarily on the number and/or density of pictorial elements in an image (Van Geert & Wagemans, 2019). In the present study, we manipulated Line Number as this changes objective complexity. We asked whether perceived (subjective) complexity as a dependent variable is modulated by objective complexity and, in addition, by Shape and Entropy, which are the focus of the present investigation. Paradoxically, although curved lines display more orientations that angular (straight) lines, they can be perceived as less complex (Bertamini et al., 2019). The relation between subjective and objective complexity is thus not straightforward and can depend on other stimulus factors, for example, symmetry (Gartus & Leder, 2017) or other factors that reflect order (Van Geert & Wagemans, 2019). By contrast, the effect of objective complexity (as an independent variable) on liking has been studied in more detail, but with heterogeneous results. People prefer an intermediate degree of physical complexity on average (Berlyne, 1974), but this general preference is subject to relatively large interindividual differences (Güclütürk et al., 2016).

Last, but not least, we wanted to contribute to the understanding of how interindividual differences relate to higher preferences of aesthetic images. To this aim, we recorded personality traits of our participants with the 50 item International Personality Item Pool (IPIP-50)* Big-Five Factor Test* (Goldberg, 1992).

In summary, we hypothesize that human observers prefer curved shape in visual stimuli where line shape is a prominent perceptual feature, and high edge-orientation entropy where lines merge into a texture. The present work contributes to our understanding of the differential role of objective stimulus features in the aesthetic evaluation of different types of stimuli.

## Methods

### Ethics Statement

The study was approved by the ethics committee of Jena University Hospital. It was carried out in accordance with the ethical guidelines of the Declaration of Helsinki. All volunteers gave their written consent before the experiment started.

### Participants

Participants were recruited by online advertisements and postings in public areas and received 8 EUR as a reward for taking part in the study. A total of 83 students (male: *N* = 23, female: *N* = 60) from diverse fields of studies participated in this experiment. All participants were between 18 and 43 years of age (*M* = 23.45 years, *SD* = 4.57). The majority came from Jena, Germany, and surrounding areas. The participants stated that they had normal or corrected-to-normal vision. In addition, we gathered the handedness of our participants via *The Edinburgh Handedness Inventory* (Oldfield, 1971; 8 left-handed, 75 right-handed).

### Edge-Orientation Entropy

Second-order entropy of edge orientations is a measure of how independent the orientations of pairs of edges are across an image (see the Introduction section). To calculate second-order entropy in the present study, we carried out a pairwise comparison of edge orientations, as originally described by Geisler et al. (2001) and modified by Redies et al. (2017). In brief, luminance edges were extracted by using 24 oriented Gabor filters, which were equally spaced and represented a full circle of orientations. The orientation of each edge element was then related to the orientation of all other edge elements in the same image by pairwise comparison. Next, we obtained histograms that indicated the weighted probability of observing an edge element at given distance *d* and direction *α* with an orientation difference *θ* for any given (reference) edge element.

As a measure for the uniformity of the obtained *θ* histograms, we calculated the Shannon entropy *H*, as follows

(1) $$H(X)=-\sum_{i=1}^{n}p(x_{i})\cdot \log_{2}p(x_{i})$$

where *X* is the *θ* histogram at distance *d* and angle *α*. The theoretical entropy maximum is about 4.585 for the 24 orientation bins of the *θ* histogram.

A high entropy value indicates a high degree of uniformity in a *θ* histogram, that is, all orientations encountered relative to the orientation of the reference edges are about equally likely to occur, and orientations are thus independent of each other. A low entropy value indicates a less uniform histogram, that is, particular orientations are more prominent than others so that the orientation of one edge predicts the orientation of other edges in the image with some nonrandom probability. To simplify the quantification of the results, we averaged entropy across direction *α*. Finally, we averaged all nonzero entropy values for distance ranges from 20 pixels to the maximal distance encountered between edge elements in the image. Edge pairs that were less than 20 pixels apart were neglected to exclude regions of local collinearity (for more details of the calculations, see Redies et al., 2017).

### Stimuli

The stimuli were constructed by systematically modifying three independent variables, which are henceforth capitalized: Shape (angular and curved), Edge-Orientation Entropy (or simply Entropy; high or low), and Line Number (5, 10, 20, and 40 line elements), resulting in 16 possible combinations of the three variables (2 × 2 × 4 design). For each combination, 10 images were generated (160 images in total).

**Figure 1.**
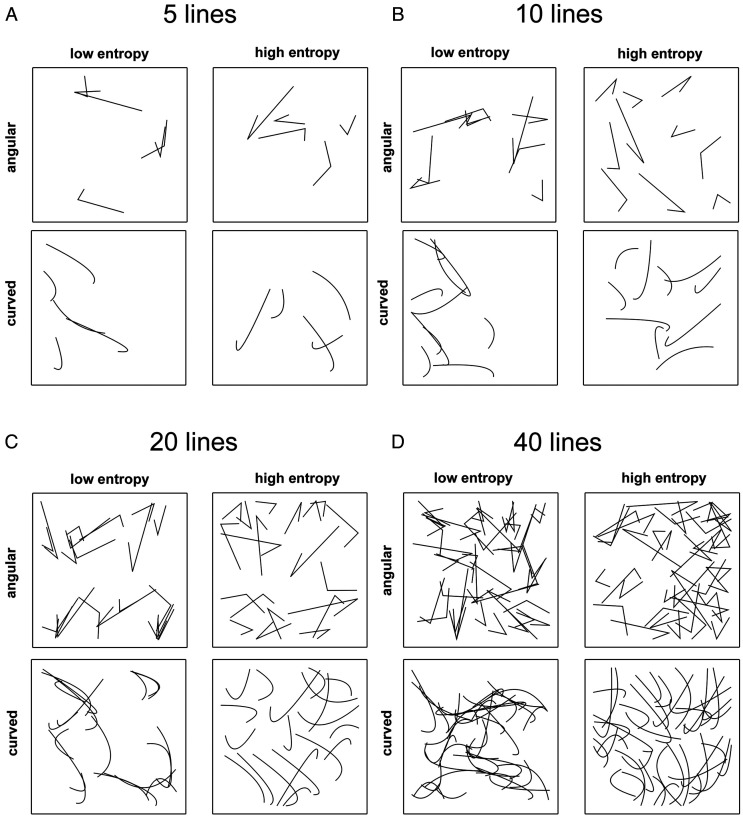
Examples of the stimuli with 5 lines (A), 10 lines (B), 20 lines (C), and 40 lines (D). In each case, examples for low and high Edge-Orientation Entropy, as well as for angular and curved Shape are shown.

To create line elements of angular or curved Shape, we followed the procedure described by Grebenkina et al. (2018), with minor modifications (for exemplary patterns, see Figure 1). In brief, we first defined sets of fixed line elements, with each consisting of three points in a plane. The three points can be connected either directly by drawing a straight line from the first point to the second point and from the second point to the third point, respectively. This procedure results in a triangular line element with one open side because there is no connection from the first point to the last point. We refer to the Shape of this type of line element as *angular*. Alternatively, the three points of each line element can be used to define a quadratic Bézier curve, which has no sharp corner. We refer to this type of line Shape as *curved*. Each set of lines contained shorter and longer lines (Figure 1). Moreover, the intervening (second) points differed in their positions relative to the first and third points.

To create images with either high or low Edge-Orientation Entropy, we chose an evolutionary approach. In a first step, we positioned each element of a line set at a random position within a defined drawing area and measured Entropy of the resulting image. A mutation of this seed image was obtained by altering the position of each of its elements with a possibility of 0.1 by translation or rotation, either of which was selected randomly. In case of a translation, an element was shifted by a random distance in each direction which was in between zero and the size of the drawing area, times a strength parameter. In case of a rotation, we rotated an element around its own center by an angle in between –*π*/2 and *π*/2, times a strength parameter. We started with a strength parameter of 0.5 and decreased it linearly until it reached 0.01 so that large changes occurred at the beginning of the process and later mutations changed the image less and less. If a mutation caused the line element to be positioned partially outside of the canvas, it was shifted back onto the drawing area. After each mutation, we measured the Entropy of the resulting image. To generate images of high Entropy, the mutated image was discarded if Entropy had decreased. If Entropy had increased, the mutated image was kept and used as the seed image for the next generation of line pattern. The evolutionary procedure was stopped when changes in Entropy became exceedingly small, as Entropy converged to a maximal value. The same procedure, but with opposite signs, was used to generate images of low Entropy.

As mentioned in the Introduction section, Shape and Entropy cannot be manipulated independently because curved lines represent more edge orientations by definition. To optimally match the automatically generated stimuli to our preconceived experimental conditions, we manually split the resulting images of both Shape groups in a low or a high Entropy condition, respectively. Average Entropy values for all conditions are given in Table 1 (see Supplementary Table 1 for statistical indices). To control for remaining Entropy differences between conditions, we entered continuously measured Entropy as a covariate into an analysis of covariance. All factors of this analysis were the same as in the design described in the Results section. This modification did not change the result pattern relevantly (Supplementary Table 2).

**Table 1.** Mean Edge-Orientation Entropy for Each Category of Pattern (2 × 2 × 4 design, *N* = 10).

	Angular	Curved
	M ± SD	M ± SD
5 Lines		
Low Entropy	3.66 ± 0.07	3.70 ± 0.10
High Entropy	3.85 ± 0.04	3.95 ± 0.04
10 Lines		
Low Entropy	3.97 ± 0.06	3.96 ± 0.08
High Entropy	4.14 ± 0.06	4.19 ± 0.05
20 Lines		
Low Entropy	3.75 ± 0.12	3.86 ± 0.10
High Entropy	4.20 ± 0.02	4.27 ± 0.02
40 Lines		
Low Entropy	3.96 ± 0.01	3.90 ± 0.08
High Entropy	4.17 ± 0.08	4.23 ± 0.08

To vary Line Number, we generated patterns that were composed of 5, 10, 20, or 40 line elements.

Stimuli and data of this study are available at the Open Science Framework (https://osf.io/zx6ph).

### Procedure

At first, participants had to fill out a questionnaire with their biographical data (age, gender, profession, and academic level). They then completed *The Edinburgh Handedness Inventory* (Oldfield, 1971). To familiarize the participants with the rating scale, they completed an introductory practice trial with five images that looked similar to those from the experimental set of stimuli but were not included in the set. The test program itself was created with PsychoPy (Peirce, 2009).

During the main experiment, a randomized stimulus set of 160 pictures was shown to the participants in three blocks, in which participants had to rate one of the dimensions *pleasing*, *complex*, or *harmonious*, respectively. We randomized the sequence of the blocks using a 3 × 3 Latin square to avoid effects of block order.

For rating, we used a numeric scale that ranged from 1 (not *pleasing/harmonious/complex*) to 6 (very *pleasing/harmonious/complex*). Participants entered their ratings by pressing keys on a standard keyboard. Every 25 pictures, participants had the possibility to take a break. The stimuli were displayed on a gray screen at a viewing distance of 70 cm that was assured by a chin rest (viewing angle, 11.06°). The pictures had a size of 500 × 500 pixels (on screen 13.5 × 13.5 cm). For each run, a fixation cross was presented first with a duration of 0.5 ms, followed by the stimulus image displayed for 2 s. The exposure time was sufficiently long to reach a stable aesthetic rating (Schwabe, 2018).

After the testing procedure, all participants had to fill out the *IPIP-50 Big-Five Factor Test* (Goldberg, 1992; Gow et al., 2005). Finally, participants were asked to give a feedback on the experimental procedure. About a third (*N* = 27) of the participants reported being influenced by their pareidolic impressions.

### Statistical Analysis

First, we analyzed the rating data by repeated-measures analyses of variance (ANOVAs) and pairwise comparisons. We tested sphericity by Mauchly’s tests. Where assumptions of sphericity were violated, we corrected degrees of freedom via the Greenhouse–Geisser procedure. The epsilon (*ϵ_GG_*) is reported where appropriate.

For pairwise comparisons, Cohen’s *d_av_* was calculated using the averaged standard deviation of the compared measurements (Lakens, 2013). In case of unplanned comparisons, corrections for multiple tests were performed following the Bonferroni–Holm approach. A 2 × 2 × 4 ANOVA was calculated with the factors Shape (angular, curved), Entropy (low, high), and Line Number (5, 10, 20, 40). In addition, we assessed whether the ratings increased linearly with increasing Line Number by polynomal contrast modeling.

As an alternative approach, we used linear mixed-effects models (lme4 library and lmerTest library in R version 4.0.0; Bates et al., 2015; Kuznetsova et al., 2017) in order to assess the effects of Line Number, Shape, and Entropy on ratings of *pleasing*, *harmonious*, and *complex* while taking into account the random variations that existed at both the participant and stimulus levels. For each rating category, separate linear mixed models were constructed using maximum likelihood estimation with incremental model complexity, and the best models were selected based on the Akaike information criterion. Following the recommendation by Barr et al. (2013), the random-effects structure was maximal, such that the random intercepts and slopes for the stimulus features within participants were included in the models unless nonconvergence was encountered. Subsequently, Shape (angular vs. smooth), Line Number (modeled as a continuous variable), and Entropy (high, low) were included as fixed effects. Based on Satterthwaite’s method to approximate degrees of freedom, we calculated *p* values for the *t* tests.

## Results

**Figure 2.**
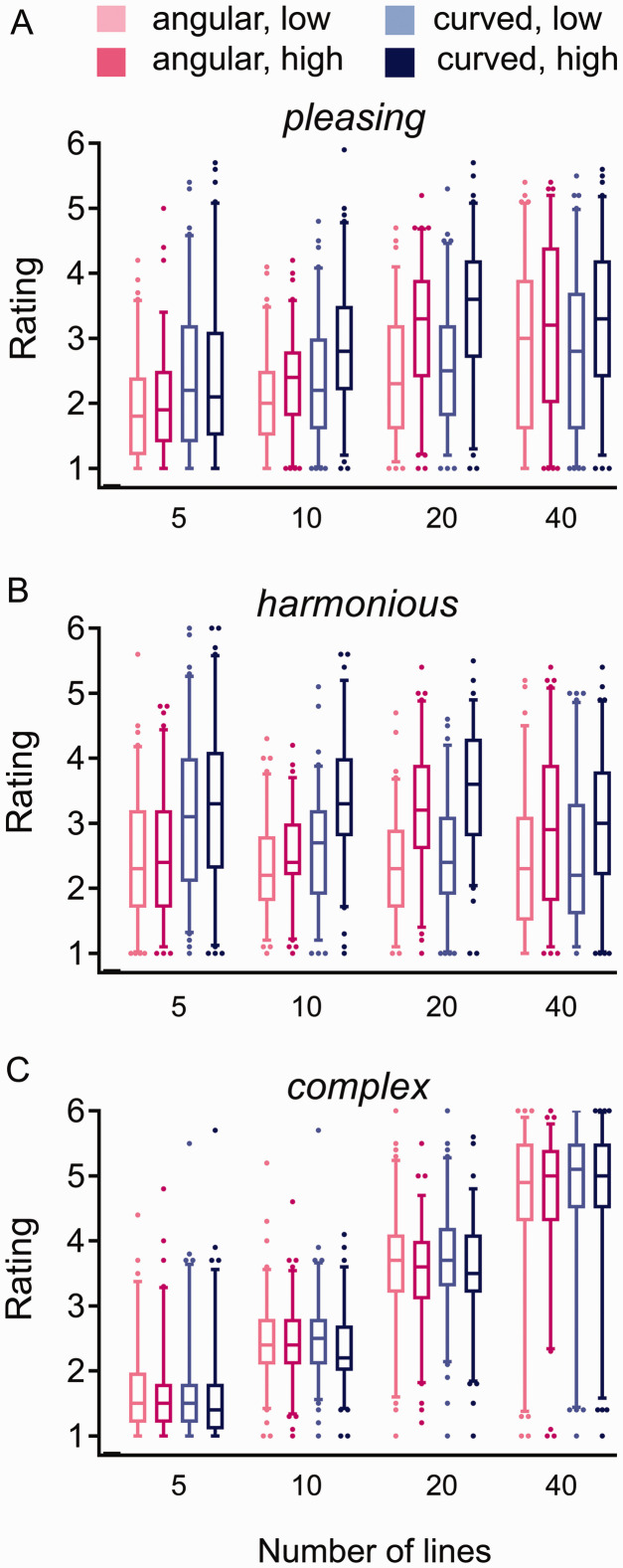
Box plots for ratings of *pleasing* (A), *harmonious* (B), and *complex* (C) for each level of Line Number and Entropy, respectively (for color coding, see top of the figure).

Figure 2 shows box plots of the rating results. For the linear mixed-effects models, a comprehensive list of statistical parameters is provided in Supplementary Table 3.

### Rating Dimension *Pleasing*

The analysis revealed a main effect of Shape for *pleasing*. Participants preferred curved over angular shapes—*M_curved_* = 2.77 ± 0.38 *SD*, *M_angular_* = 2.50 ± 0.47 *SD*, *F*(1, 82) = 12.56, *p* = .001, $\eta_{p}^{2}$ = 0.13, *d* = 0.37, confidence interval [CI] [0.20, 0.51]. There was also a main effect of Entropy, *F*(1, 82) = 66.35, *p* < .001, $\eta_{p}^{2}$ = 0.45, *d* = –0.60, CI [0.44, 0.74]. On average, participants liked images with high Entropy better than those with lower Entropy (*M_high_* = 2.84 ± 0.48 *SD*, *M_low_* = 2.43 ± 0.29 *SD*). Furthermore, Line Number had an impact on participants’ *pleasing* ratings, *F*(3, 246) = 24.20, *p* < .001, $\eta_{p}^{2}$ = 0.23, with numerically increasing ratings for images with more lines—$M_{5\;\mathrm{lines}}$ = 2.20 ± 0.24 *SD*, $M_{10\;\mathrm{lines}}$ = 2.40 ± 0.29 *SD*, $M_{20\;\mathrm{lines}}$ = 2.87 ± 0.39 *SD*, $M_{40\;\mathrm{lines}}$ = 3.05 ± 0.24 *SD*; 5 versus 10: *t*(82) = –3.35, *p* =.01, *d* = –0.23, CI [–0.43, –0.08]; 10 versus 20 lines: *t*(82) = –6.37, *p* < .01, *d* = –0.64, CI [–0.79, –0.47]. Only the rating difference between 20 and 40 lines did not reach significance (*p* > .05). Polynomial contrast modeling revealed that ratings of *pleasing* increased linearly with Line Number increasing from 5 to 40 lines, *F*(1, 82) = 25.01, *p* < .001, $\eta_{p}^{2}$ = .234.

These main effects, however, were qualified by the interactions of Shape × Line Number, *F*(3, 246) = 23.42, p<.001, $\eta_{p}^{2}$ =.22, *ϵ_GG_* = 0.74. In line with our hypotheses, paired *t* tests revealed a significantly smaller preference for curved stimuli for 40 lines compared with images with fewer lines—5 lines: *t*(82) = –6.50, *p* < .001, *d* = –0.47, CI [–0.65, –0.23]; 10 lines: *t*(82) = –4.95, p<.001, *d* = –0.50, CI [–0.64, –0.30]; 20 lines: *t*(82) = –2.52, *p* =.013, *d* = –0.26, CI [–0.44, –0.07]; 40 lines: *t*(82) =.04, *p* >.05, *d* = 0.00, CI [–0.28, 0.25]. Generally, this Shape effect decreases with increasing Line Number and is not significant for 40 lines. In other words, people prefer curved over angular shapes only for images with few lines (20 lines and less). Paired *t* tests revealed a significantly reduced effect of Shape for 20 versus 10 lines, *t*(82) = –2.94, *p* =.004, *d* = –0.22, CI [–0.37, –0.05], and for 40 versus 20 lines, *t*(82) = –4.15, *p* < .01, *d* = –0.26, CI [–0.44, –0.08].

Furthermore, the interaction of Entropy × Line Number was significant in this analysis, *F*(3, 246) = 30.51, p<.001, $\eta_{p}^{2}$ = 0.27, *ϵ_GG_* = 0.73. Contrary to the Shape effect, the benefit for high versus low Entropy is only significant for images with more than 5 lines—5 lines: *t*(82) = –1.75, *p* =.84, *d* = –0.06, CI [–0.26, 0.15]; 10 lines: *t*(82) = –7.19, *p* < .01, *d* = –0.51, CI [–0.66, –0.34]; 20 lines: *t*(82) = –7.53, *p* < .01, *d* = –0.83, CI [–1.01, –0.63]; 40 lines: *t*(82) = –7.13, *p* < .01, *d* = –0.39, CI [–0.65, –0.14]). Paired *t* tests revealed a significantly smaller effect of Entropy for 5 compared with all Line Numbers—all *t*(82) < –5.47, all *p* < .001, all *d* < –0.80.

There was also an interaction of Shape × Entropy, *F*(1, 82) = 9.12, *p* =.003, $\eta_{p}^{2}$ = 0.10, *ϵ_GG_* = 1.0. The Entropy effect in the direction described earlier is generally larger for curved stimuli compared with angular images, *t*(82) = –3.02, *p* ≤.003, *d* = –0.47, CI [–0.62, –0.31]. However, this difference further depends on Line Number, as indicated by a three-way interaction of Shape × Entropy × Line Number, *F*(3, 246) = 5.33, *p* = .001, $\eta_{p}^{2}$ = 0.06, *ϵ_GG_* = 0.934. We analyzed this interaction by performing separate ANOVAs for all levels of Line Number with the factors Entropy and Shape, and comparing their result patterns. For 5 lines, there was a significant effect of Shape only, *F*(1, 82) = 42.25, p<.001, $\eta_{p}^{2}$ = 0.34, *ϵ_GG_* = 1.0, and none for Entropy or any interaction (p>.05). For 10 lines, both main effects, Shape, *F*(1, 82) = 24.48, p<.001, $\eta_{p}^{2}$ = 0.23, *ϵ_GG_* = 1.0, and Entropy, *F*(1, 82) = 51.73, p<.001, $\eta_{p}^{2}$ = 0.39, *ϵ_GG_* = 1.0, were present as well as their interaction, *F*(1, 82) = 18.86, p<.001, $\eta_{p}^{2}$ = 0.18, *ϵ_GG_* = 1.0. For 20 lines, significant effects for Shape, *F*(1, 82) = 6.38, *p* = .013, $\eta_{p}^{2}$ = 0.07, *ϵ_GG_* = 1.0, and Entropy, *F*(1, 82) = 56.73, p<.001, $\eta_{p}^{2}$ = 0.41, *ϵ_GG_* = 1.0, were observed, but not for their interaction. For 40 lines, there was a strong effect for Entropy, *F*(1, 82) = 50.88, p<.001, $\eta_{p}^{2}$ = 0.38, *ϵ_GG_* = 1.0, no effect of Shape, and only a modest interaction that did not survive Bonferroni correction (critical alpha =.013).

**Figure 3.**
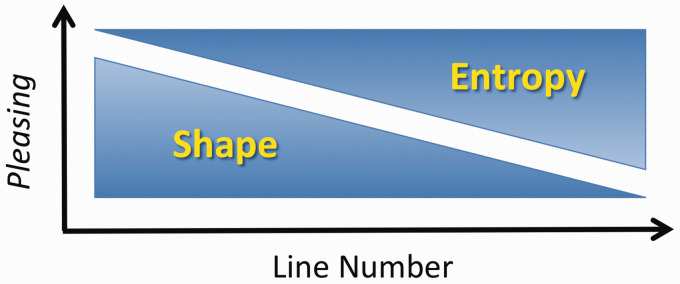
Hypothesis of how the rating of *pleasing* depends on Line Number. With increasing Line Number, the influence of Shape effects, such as the preference for curvature, decreases, whereas the influence of Entropy effects, such as the preference for higher Entropy, increases.

Taken together, the influence of Shape was only seen for images with few lines, while the influence of Entropy was stronger for images with many lines. These effects remain stable even when continuous Entropy is taken into account (see the Methods section and Supplementary Table 2 for details). Figure 3 shows a simplified hypothetical model of the differential effect of Shape and Entropy on *pleasing* ratings.

The linear mixed-effects model analysis (Supplementary Table 3) revealed significant main effects for Shape, Line Number, and Entropy, as well as for all two-way interactions. Main effects were strongest and positive for Line Number, *β* = 0.035, *SE* = 0.0013, *t*(13030) = 27.0, p<.0001, and Entropy, *β* = 0.163, *SE* = 0.0610, *t*(179.6) = 2.67, p<.01. Moreover, curved Shape was liked more than angular Shape, *β* = 0.597, *SE* = 0.083, *t*(121.4) = 7.20, p<.0001, but this effect decreased with increasing Line Number (interaction for Shape × Line Number); *β* = 0.0134, *SE* = 0.0015, *t*(13030) = 8.94, p<.0001. There was also an interaction for Shape × Entropy, *β* = 0.154, *SE* = 0.0402, *t*(13030) = 3.83, p<.001. Curved Shape was a stronger predictor when Entropy was high. Also, the predictive influence of Entropy was stronger when Line Number was higher (interaction for Entropy × Line Number); *β* = 0.0092, *SE* = 0.0015, *t*(13030) = 6.12, p<.0001.

The linear mixed-effects model accounted for 8.4% of the variance without the random-effect structure ($R^{2}m$) and for 35.6% when it was included ($R^{2}c$). These findings largely confirm the results from the ANOVA analysis with respect to the influence of Shape, Line Number, and Entropy on *pleasing* ratings, as well as their two-way interactions. The three-way interaction of Shape × Line Number × Entropy did not reach significance in the mixed-effects analysis.

### Rating Dimension *Harmonious*

The pattern of ANOVA results for the rating of *harmonious* was similar to *pleasing* ratings in general. There was a prominent main effect of Shape, *F*(1, 82) = 29.06, p<.001, $\eta_{p}^{2}$ = 0.26, *d* = –0.60. Curved stimuli were rated as more *harmonious* than angular ones (*M_curved_* = 3.00 ± 0.66 *SD*, *M_angular_* = 2.59 ± 0.70 *SD*). There was also a main effect of Entropy, *F*(1, 82) = 68.55, *p* <.001, $\eta_{p}^{2}$ = 0.46, *d* = 0.79, with high-Entropy images being rated as more *harmonious* (*M_high_* = 3.01 ± 0.36 *SD*, *M_low_* = 2.54 ± 0.24 *SD*). No main effect of Line Number was observed in the ANOVA. Also, we found no evidence from polynomial contrast modeling that participants’ rating of *harmonious* increased linearly with Line Number increasing from 5 to 40 lines, *F*(1, 82) = 0.12, *p* < .733, $\eta_{p}^{2}$ =.001.

These main effects were qualified by the interaction of Shape × Line Number, *F*(3, 246) = 43.18, p<.001, $\eta_{p}^{2}$ = 0.35, *ϵ_GG_* = 0.72. The Shape effect decreases with increasing Line Number, with a significant decline between 10 and 20 lines, *t*(82) = –7.99, p<.001, *d* = –.058, and between 20 and 40 lines, *t*(82) = –2.91, *p*=.005, *d* = 0.55.

Also similar to the *pleasing* rating, we found an interaction of Entropy × Line Number, *F*(3, 246) = 48.00, p<.001, $\eta_{p}^{2}$ = 0.37, *ϵ_GG_* = 0.73. The effect of Entropy increased between 5 and 10 lines, *t*(82) = –7.5, p<.001, *d* = –0.87, as well as between 10 and 20 lines, *t*(82) = –6.3, p<.001, *d* = –0.59, before decreasing again between 20 and 40 lines, *t*(82) = –6.7, p<.001, *d* = 0.57. Participants’ judgment of *harmonious* depended more strongly on Entropy for images with more than five lines.

We also found a significant interaction of Shape × Entropy, *F*(1, 82) = 12.00, *p* =.001, $\eta_{p}^{2}$ = 0.13, $\eta_{p}^{2}$ = 1.00. This indicated a generally larger benefit of high Entropy in ratings of curved compared with angular stimuli, *t*(82) = –3.5, *p* = .001, *d* = –0.32, CI [–0.41, –0.16]. Post hoc inspection of a significant three-way interaction, *F*(3, 246) = 11.00, p<.001, $\eta_{p}^{2}$ = 0.12, of this effect with Line Number showed, however, that the effect was significant only for stimuli with 10 lines, *t*(82) = –6.32, p<.001, *d* = –0.83, CI [–0.93, –0.64].

Like in the ANOVA analysis, we obtained a generally similar result pattern for the ratings of *pleasing* and *harmonious* in the mixed-effects model analysis (Supplementary Table 3). Again, there were main effects for Shape, Line Number, and Entropy. The main effects were strongly positive for Entropy, *β* = 0.189, *SE* = 0.0770, *t*(198.8) = 2.46, *p* =.015, and curved Shape was preferred over angular Shape, *β* = 0.908, *SE* = 0.0879, *t*(156.3) = 10.33, p<.0001. The mixed-effects analysis revealed an effect of Line Number that was not seen with the ANOVA. *Harmonious* ratings were higher with increasing Line Number, *β* = 0.013, *SE* = 0.0015, *t*(13030) = 8.28, p<.0001. Like in the ANOVA analysis, all two-way interactions were significant (Supplementary Table 3). There was also a significant three-way interaction of Shape × Line Number × Entropy. The mixed-effects model accounted for 6.3% of the variance without the random-effect structure ($R^{2}m$) and for 31.1% when it was included ($R^{2}c$).

### Rating Dimension *Complex*

For ratings of *complex*, there was a main effect of Line Number—$M_{5\;\mathrm{lines}}$ = 1.67 ± 0.68 *SD*, $M_{10\;\mathrm{lines}}$ = 2.45 ± 0.52 *SD*, $M_{20\;\mathrm{lines}}$ = 3.61 ± 0.08 *SD*, and $M_{40\;\mathrm{lines}}$ = 4.68 ± 0.12 *SD*, *F*(3, 246) = 299.42, p<.001, $\eta_{p}^{2}$ = 0.79—with higher ratings of *complex* for images with more lines—5 vs. 10 lines: *t*(82) = –13.59, p<.001, *d* = –1.29, CI [–1.44, –1.18]; 10 vs. 20 lines: *t*(82) = –17.57, p<.001, *d* = –1.81, CI [–1.93, –1.65]; and 20 vs. 40 lines: *t*(82) = –15.35, p<.001, *d* = –1.14, CI [–1.30, –0.09]. Polynomial contrast modeling showed that participants’ rating of *complex* increased linearly with Line Number increasing from 5 to 40 lines, *F*(1, 82) = 321.74, p<.001, $\eta_{p}^{2}$ =.797.

Moreover, the ANOVA indicated a two-way interaction of Shape × Entropy, *F*(1, 82) = 82.00, *p* =.045, $\eta_{p}^{2}$ = 0.05. Participants rated curved stimuli with high Entropy as less *complex*. This interaction, however, was not stable in post hoc comparisons (all p>.144, all d< 0.14). In contrast to ratings of *pleasing* and *harmonious*, there were no significant differences for the effect of Entropy and Shape (all p>.05).

The linear mixed-effects analysis revealed a main effect of Line Number only. As expected and already seen in the ANOVA, images with higher Line Number were rated as more *complex*, *β* = 0.0836, *SE* = 0.0011, *t*(13030) = 73.4, p<.0001. There was a significant two-way interaction for Line Number × Entropy, *β* = 0.0031, *SE* = 0.0013, *t*(13030) = 2.35, *p* = .019. Images of high Entropy were rated as less *complex* when Line Number was low. We also observed a trend for an interaction of Shape × Entropy, *β* = 0.0657, *SE* = 0.0353, *t*(13030) = 1.86, *p* = .063, but the three-way interaction was not significant (Supplementary Table 3). The mixed-effects model accounted for 47.8% of the variance without the random-effect structure ($R^{2}m$) and for 60.3% when it was included ($R^{2}c$).

### IPIP-50 Big-Five-Factor Test

The analysis of personality trait effects was carried out with a sample of 78 participants. Five people had to be excluded because they did not complete the questionnaire. Upon inspection of the trait distributions of all Big-Five dimensions, we noticed that the variance in our sample was comparatively low across all personality dimensions (see Supplementary Figure 1). In addition, the distribution of the traits for Agreeableness, Conscientiousness, and Openness/Intellect was shifted toward high values. For example, only two participants scored lower in Openness/Intellect than the scale midpoint of 30 points. We thus concluded that our sample of participants was not well suited to analyze the influence of personality traits on the ratings. Nevertheless, we share the original data for inclusion in possible future studies (Open Science Framework: https://osf.io/zx6ph).

## Discussion

In the present study, we investigated the relation between subjective aesthetic ratings and the shape and spatial distribution of oriented line elements in visual stimuli. In designing the experiment, we took up an observation by Grebenkina et al. (2018) who showed that participants prefer complex abstract line patterns, in which the edge orientations are distributed independently across the stimulus. As a measure of how independent the distribution of edge orientations is, the authors calculated second-order Shannon entropy of edge orientations (Geisler et al., 2001) for each image. In their study, edge-orientation entropy turned out to be a more powerful predictor for aesthetics ratings of pleasing and interesting than shape (curved or angular). This finding is at odds with the general preference for curved over angular shapes that was found robustly in many other studies (see the Introduction section). Speculating about the origin of this discrepancy, Grebenkina et al. (2018) pointed out that their stimuli consisted of many lines, thus forming a texture, in which the shape of individual lines was no longer a perceptually prominent feature. The aim of the present study was to test this notion by systematically reducing the number of lines in the stimuli until the shape of individual lines became perceptually more conspicuous.

### Stimulus-Dependent Effects of Curvilinear Shape and Edge-Orientation Entropy

The present results support the idea that people prefer curved stimuli over angular ones overall. Specifically, participants rated curved stimuli as more *pleasing* and *harmonious* than the angular stimuli (Figure 2A and B). At the same time, participants also rated stimuli more highly if they showed higher Edge-Orientation Entropy. Shape of the lines was a stronger predictor for ratings of *pleasing* and *harmonious* when participants viewed a few lines only (e.g., five lines). Vice versa, Edge-Orientation Entropy was a stronger predictor of the ratings when many lines merged into a texture (e.g., with 40 lines; Figure 2A and B; for a schematic summary diagram, see Figure 3). These results imply that the two determinants of visual preference play a different role in shapes and textures, respectively. Our findings thus resolve the apparent incongruity between the results by Grebenkina et al. (2018) and the widely reported preference for curved over angular shapes.

Differences in the perception between shapes and textures have been subject of many investigations before. A classic example is the effect by Galli and Zama (1931) who explored a triangle with and without a surrounding grating, which consisted of parallel lines. If the triangle was presented in isolation, all three sides were perceived as equally salient. If a line grating surrounded the triangle, the side of the triangle that ran in parallel to the grating was no longer perceived as a part of the triangle. Instead, the line was perceived as one of the many parallel lines that formed the grating texture. This and other observations indicate that the human visual system processes the shape of isolated contours differently from the shape of elements in textures (for a review, see Gheorghiu et al., 2014).

### Aesthetic Preferences and the Processing of Shape and Texture

Effects of surrounding texture on shape perception may be explained by neurophysiological principles of receptive fields. The classical receptive field (CRF) of a visual cortical (V1) neuron is defined as the region that responds directly to retinal stimulation. Stimulating the area beyond the CRF does not usually elicit a response. However, such a surrounding stimulation can modulate the responses elicited by CRF stimulation (Maffei & Fiorentini, 1976). The modulatory surrounding region has been called the extraclassical receptive field (ERF) or association field (Field et al., 1993; for a review, see Spillmann et al., 2015). Typically, if iso-oriented stimuli are used, ERF stimulation suppresses CRF responses. With stimuli of different orientations, ERF stimulation enhances CRF responses. It has been proposed that, in general, the presence of structural regularities in an image decreases activity in V1 neurons (Rao & Ballard, 1999). Physiological substrates of such ERF modulations may be feedback connections from higher cortical regions (Rao & Ballard, 1999) or long-range horizontal connections in visual cortex (Hunt et al., 2011). Considering our stimuli, which consist of oriented lines or edges, participants prefer images with high Entropy, that is, with low regularities in edge orientations. As Entropy decreases, regularities in edge orientations, such as collinearity and parallelism, become more prominent (see examples in Figure 1). In view of these findings, we speculate that ERF effects on V1 neuronal responses may mediate the aesthetic ratings for images of low and high Edge-Orientation Entropy, respectively.

Neural correlates of shape and texture perception have been allocated to different brain regions, which interact with each other (Gheorghiu et al., 2014). While texture processing is thought to take place predominantly in V1 (see earlier), shape processing takes place mostly in extrastriate cortical regions (Dumoulin et al., 2008; Vinberg & Grill-Spector, 2008).

### Image Properties and Aesthetic Preference of Different Types of Visual Stimuli

The present study reveals differences in the image features that predict aesthetic ratings of *pleasing* and *harmonious* in two types of stimuli, lines and textures (Figure 3). Other types of visual stimuli also differ in the image features that carry information about visual preference. A particularly striking example is portrait paintings (Redies et al., 2015). On the one hand, viewers can rate the attractiveness of the face depicted in the portrait. On the other hand, viewers can evaluate the artistic beauty of the painting composition. Based on an adaptation study, Schulz and Hayn-Leichsenring (2017) suggested that the two types of ratings are mediated by different neural mechanisms. Mather (2020) measured image statistics (Fourier spectral slope, fractal dimension, and luminance entropy) in different painting genres; he found that the values for the three measures varied across genres (for a similar study, see Hayn-Leichsenring et al., 2017). Differences in statistical image properties can also be used to identify artistic styles automatically (Hughes et al., 2011; Wallraven et al., 2009). However, there is also some evidence that specific stimulus features can be shared between aesthetic stimuli, such as artworks of different cultural provenance (see the Introduction section). For example, artworks of Western, Islamic, and Chinese provenance display a similar degree of edge-orientation entropy (Redies et al., 2017) and share regularities in the variances of features that were derived from a model of low-level visual processing (Brachmann et al., 2017). To determine to what extent particular perceptual features play a role in universal and/or domain-specific aspects of aesthetic evaluations will require more detailed studies in the future.

### Ratings of *Complex*

As expected, the ratings of *complex* increased with increasing Line Number (Figure 2C). Interestingly, participants tended to rate curved stimuli with high Entropy as subjectively less *complex*. This trend seems paradoxical because, objectively, curved lines display more orientations than angular (straight) lines and should therefore be perceived as more complex, as discussed in Bertamini et al. (2019). The authors showed that, in addition, curved shapes are processed faster than angular ones. To explain this apparent paradox, Bertamini et al. argued that curved shapes are more prevalent in the natural environment, to which the human visual system is adapted. As a result of this adaptation, curved shapes may be processed more efficiently. In turn, more efficient processing may cause stimuli to be perceived as more pleasing (Redies, 2007; Renoult et al., 2016). To find out whether our present findings can be generalized to other stimuli, more experiments with carefully controlled stimuli are needed.

### Interindividual Differences

Despite the general similarities in aesthetic judgments across participants, individual beholders can respond differently to image properties. Examples for such interindividual differences in aesthetic judgments have been described for the perception of color and self-similarity (Mallon et al., 2014) as well as complexity (Bies et al., 2016; Spehar et al., 2015). Cotter et al. (2017) showed that Openness/Intellect is a personality factor that correlates with greater liking for curvature. In the present study, we wanted to confirm this previous finding. However, our sample of participants was too small and not well suited to inspect such differences. Unfortunately, participants scored relatively high in Openness/Intellect in absolute terms and did not strongly differ in this trait (Supplementary Figure 1). Moreover, the variance in our sample of participants was comparatively low across all personality domains (Supplementary Figure 1). As a result, the differences between high and low scoring groups were generally moderate in size at best. Further research with greater power may well result in better insight into the inner workings of this preference.

### Limitations and Comparison with Other Studies

To better control the experimental parameters, the present study was carried out in a laboratory setting with artificial abstract stimuli. This approach has the advantage that it minimizes the influence of possible confounding factors, such as color, stimulus contrast, viewing distance, or image content on the ratings; it thus guarantees high reproducibility and internal validity. Although our approach minimizes confounding factors in general, we cannot exclude that we introduced secondary effects that may have contributed to the aesthetic ratings. For example, the experimental modulation of Entropy has an obvious effect on the arrangement of the line elements, which overlapped and crossed each other more frequently when Entropy was minimized (Figure 1). Moreover, with maximized Entropy, lines crossed each other more often at right angles and the spacing between lines was more regular. The effect of such secondary structural differences on the ratings should be studied in more detail in the future. Moreover, about one third of the participants reported pareidolic impressions, that is, they saw imaginated objects when viewing the abstract patterns. The role of such phenomena in aesthetic perception remains to be studied in more detail.

The disadvantage of our well-controlled approach is that results cannot necessarily be extrapolated to complex natural stimuli in everyday life, or to artworks in a museum environment (Tschacher et al., 2012). Also, in more complex natural stimuli, the observed effects may be modulated or even abolished by other stimulus properties that affect visual preference (Makin, 2017). However, despite this low external validity, there are hints in the literature that the present results do relate to less artificial situations. First, preferences for curved objects have been demonstrated also for real objects (e.g., Bar & Neta, 2006). Second, a preference for curvature has been demonstrated across different cultures (Gómez-Puerto et al., 2018) and even in great apes (Munar et al., 2015). Third, it has been reported that a large subset of traditional artworks of diverse cultural backgrounds share high edge-orientation entropy (Brachmann et al., 2017). Fourth, high edge-orientation entropy also mediates preference of everyday displays, such as CD album covers and architecture (Grebenkina et al., 2018; Thömmes & Hübner, 2018). Thus, although our results from simple line elements cannot be generalized readily to more complex stimuli, they are in line with previous findings.

## Supplemental Material

sj-pdf-1-ipe-10.1177_2041669520950749 - Supplemental material for Aesthetic Perception of Line Patterns: Effect of Edge-Orientation Entropy and Curvilinear Shape Click here for additional data file. Supplemental material, sj-pdf-1-ipe-10.1177_2041669520950749 for Aesthetic Perception of Line Patterns: Effect of Edge-Orientation Entropy and Curvilinear Shape by Sarah Stanischewski, Carolin S. Altmann, Anselm Brachmann and Christoph Redies in i-Perception
