# Supplementary material for: Aesthetic Perception of Line Patterns: Effect of Edge-Orientation Entropy and Curvilinear Shape
Source: Iperception. 2020 Sep 30;11(5):2041669520950749. doi: 10.1177/2041669520950749 (PMC7533941; doi:10.1177/2041669520950749)
Supplement: sj-pdf-1-ipe-10.1177_2041669520950749 - Supplemental material for Aesthetic Perception of Line Patterns: Effect of Edge-Orientation Entropy and Curvilinear Shape [file sj-pdf-1-ipe-10.1177_2041669520950749.pdf]

SUPPLEMENTARY MATERIAL

Supplementary Table 1. Statistical indices for the ANOVA testing for differences in continuous Edge-Orientation Entropy between experimental conditions. Entropy served as the dependent variable. Factors and factor levels were identical to the experimental conditions used in the rating experiment. Note that all effects that include Shape, are of modest size only, thus restricting inherent interdepencies at least partly.

	F-value (*df*)	*p*-value	Partial eta squared (η^2_p)
Shape	$F(1, 144) = 12.56$	$p = .001$	$\eta^2_p = 0.08$
Line Number	$F(3, 144) = 125.04$	$p < .001$	$\eta^2_p = 0.72$
Entropy	$F(1, 144) = 571.87$	$p < .001$	$\eta^2_p = 0.80$
Shape × Line Number	$F(3, 144) = 3.30$	$p = .022$	$\eta^2_p = 0.06$
Shape × Entropy	$F(1, 144) = 3.76$	$p = .054$	$\eta^2_p = 0.03$
Line Number × Entropy	$F(3, 144) = 18.48$	$p < .001$	$\eta^2_p = 0.28$
Shape × Line Number × Entropy	$F(3, 144) = 1.56$	$p = .201$	$\eta^2_p = 0.03$

Supplementary Table 2. ANCOVA results with *pleasing* as the dependent variable. The factors and factor levels were the same as in the main experiment. Edge-Orientation Entropy = continuous Entropy value. Entropy* = categorial Edge-Orientation Entropy split into low and high factor levels. The inclusion of the continuous Entropy values in the ANCOVA showed no significant influence on the pattern of results.

Covariate:	*F*-value (*df*)	*p*-value	Partial eta squared (η^2_p)
Edge-Orientation Entropy	$F(1, 144) = 5.03$	$p = .027$	$\eta^2_p = 0.03$
Factors:			
Shape	$F(1, 144) = 54.12$	$p < .001$	$\eta^2_p = 0.28$
Line Number	$F(3, 144) = 91.91$	$p < .001$	$\eta^2_p = 0.66$
Entropy*	$F(1, 144) = 13.89$	$p < .001$	$\eta^2_p = 0.09$
Shape × Line Number	$F(3, 144) = 9.58$	$p < .001$	$\eta^2_p = 0.17$
Shape × Entropy*	$F(1, 144) = 4.00$	$p = .047$	$\eta^2_p = 0.03$
Line Number × Entropy*	$F(3, 144) = 11.55$	$p < .001$	$\eta^2_p = 0.20$
Shape × Line Number × Entropy*	$F(3, 144) =1.62$	$p = .186$	$\eta^2_p = 0.03$

Supplementary Table 3. Statistical indices for the fixed effects of the linear mixed-effects models for the ratings of *pleasing*, *harmonious* and *complex*.

pleasing

Fixed Effect	β	*SE*	*df*	*t*-value	*p*-value
Intercept	2.010	0.0855	101.3	23.51	< .0001
Shape	0.597	0.0829	121.4	7.20	< .0001
Line Number	0.035	0.0013	13030	27.00	< .0001
Entropy	0.163	0.0610	179.6	2.67	= .008
Shape x Line Number	0.0134	0.0015	13030	8.94	< .0001
Shape x Entropy	0.154	0.0402	13030	3.83	< .001
Line Number x Entropy	0.0092	0.0015	13030	6.12	< .0001

harmonious

Fixed Effect	β	*SE*	*df*	*t*-value	*p*-value
Intercept	2.560	0.0921	105.0	27.79	< .0001
Shape	0.908	0.0879	156.3	10.33	< .0001
Line Number	0.0128	0.0015	13030	8.28	< .0001
Entropy	0.189	0.0770	198.8	2.46	= .015
Shape x Line Number	0.0216	0.0022	13030	9.93	< .0001
Shape x Entropy	0.332	0.0711	13030	4.67	< .0001
Line Number x Entropy	0.0123	0.0022	13030	5.64	< .0001
Shape x Line Number x Entropy	0.0076	0.0031	13030	2.45	= .014

complex

Fixed Effect	β	*SE*	*df*	*t*-value	*p*-value
Intercept	1.50	0.0681	106.0	22.04	< .0001
Shape	-0.040	0.0444	290.1	-0.90	= .368
Line Number	0.0836	0.0011	13030	73.37	< .0001
Entropy	0.0728	0.0542	176.0	1.34	= .181
Shape x Line Number	0.0020	0.0013	13030	1.53	= .127
Shape x Entropy	0.0657	0.0353	13030	1.86	= .063
Line Number x Entropy	0.0031	0.0013	13030	2.35	= .019

SUPPLEMENTARY MATERIAL

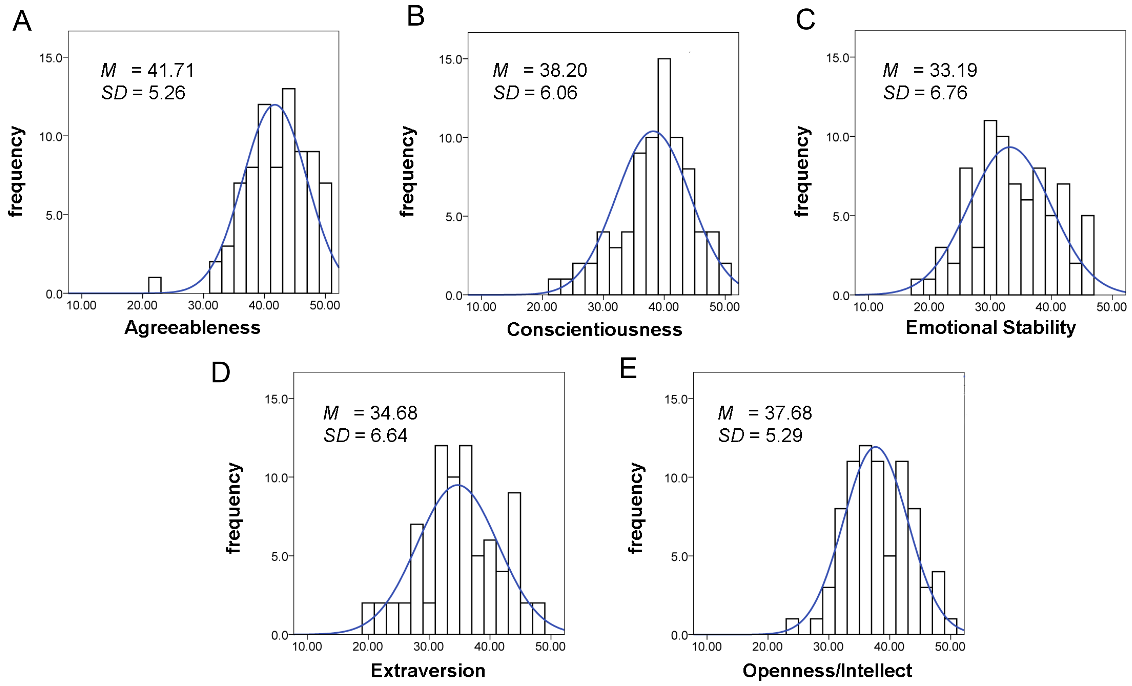

Supplementary Figure 1: Histograms of the results of the IPIP-50 Big-Five Factor test. The variance in our sample was comparatively low across the personality domains Agreeableness (A), Conscientiousness (B), Emotional Stability (C), Extraversion (D) and Openness/Intellect (E).
